# Expanding frontiers in weight-control research explored by young investigators

**DOI:** 10.1007/s12576-016-0495-7

**Published:** 2016-10-11

**Authors:** Yoshiro Ishimaru, Chisayo Kozuka, Kenichiro Nakajima, Tsutomu Sasaki

**Affiliations:** 1 Department of Applied Biological Chemistry, Graduate School of Agricultural and Life Sciences, The University of Tokyo, 1-1-1 Yayoi, Bunkyo-ku, Tokyo, 113-8657 Japan; 2 Division of Endocrinology, Diabetes and Metabolism, Hematology, Rheumatology (Second Department of Internal Medicine), Graduate School of Medicine, University of the Ryukyus, Okinawa, Japan; 3 Laboratory for Metabolic Signaling. Institute for Molecular and Cellular Regulation, Gunma University, Maebashi, Japan

**Keywords:** Type 2 immune response, γ-Oryzanol, Appetite, DREADD, Anorexia

## Abstract

At the 93rd annual meeting of the Physiological Society of Japan, a symposium entitled “Expanding frontiers in weight-control research explored by young investigators” was organized. The latest research on weight control was presented by young up-and-coming investigators. The symposium consisted of the following presentations: Gastrointestinal brush cells, immunity, and energy homeostasis; Impact of a brown rice-derived bioactive product on feeding regulation and fuel metabolism; A novel G protein-coupled receptor-regulated neuronal signaling pathway triggers sustained orexigenic effects; and NMDA receptor co-agonist d-serine regulates food preference. These four talks presented at the symposium were summarized as a series of short reviews in this review.

## Main text

### Gastrointestinal brush cells, immunity, and energy homeostasis (by Yoshiro Ishimaru)

The intestinal epithelium has at least two critical functions: absorption of nutrients and defense against pathogens and parasites. There are four major cell types in the small intestine: enterocytes, Goblet, Paneth, and enteroendocrine cells [1]. Enterocytes absorb nutrients, water, and electrolytes, whereas Goblet, Paneth, and enteroendocrine cells secrete mucus, peptides that provide defense against microbes, and gastrointestinal hormones that regulate intestinal function and communication with other organs. In addition to these cell types, brush cells (also referred to as tuft cells or caveolated cells) constitute a minor fraction (0.4 %) of the adult mouse intestinal epithelium [2, 3], and they are also found in the trachea, salivary glands, and stomach. Brush cells are characterized by their unique morphology, with an extensively developed cytoplasmic tubulovesicular system and an apical bundle of microfilaments connected to a tuft of long and thick microvilli projecting into the lumen [4]. These cells express transient receptor potential melastatin 5 (*Trpm5*), doublecortin-like kinase 1 (*Dclk1*), and choline acetyltransferase [2, 5–8]. Although brush cells were discovered more than 50 years ago [9], the mechanisms of differentiation and the function of these cells have remained elusive.

The *Skn*-*1* (also known as *Pou2f3*) gene, which encodes the POU homeodomain transcription factor, was originally identified as a regulator of the differentiation of epidermal keratinocytes [10, 11]. Subsequently, it was reported that *Skn*-*1a* is also expressed in sweet, umami (savory), and bitter-sensing taste cells (referred to as type II taste cells) in the taste buds, in solitary chemosensory cells in the nasal respiratory epithelium, and in microvillus cells in the main olfactory epithelium and is required for the differentiation of these types of *Trpm5*-expressing cells [12–14]. Recently, we and another group demonstrated that neither *Dclk1* nor *Trpm5* is expressed in the gastrointestinal (GI) tract of *Skn*-*1* knockout (KO) mice [15, 16]. These results demonstrate that brush cells are completely abolished in *Skn*-*1* KO mice and that Skn-1a is a crucial transcription factor for generating brush cells in the GI tract.

**Fig. 1:**
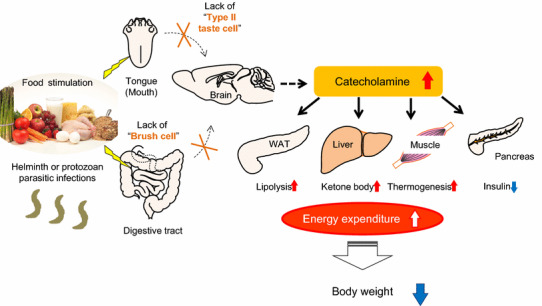
Schematic representation of the presumed metabolic pathways originating from brush cells and type II taste cells in the GI tract. Food components and digested nutrients are detected by the tongue and GI tract. In *Skn*-*1* KO mice, the lack of type II taste cells and brush cells results in increased catecholamine secretion. Lipolysis in WAT, serum levels of total ketone bodies, and thermogenesis in muscle are increased, whereas insulin secretion from the pancreas is reduced. Consequently, *Skn*-*1* KO mice have reduced body weight with lower body fat percentage due to higher energy expenditure. In addition, brush cells play a pivotal role in the initiation of type 2 immune responses induced by helminth and protozoan parasitic infections. Portions of this figure were modified from Fig. 6 in Ushiama et al. [16]

To examine the effect on energy homeostasis of eliminating type II taste cells and brush cells in the GI tract, we characterized the metabolic phenotypes of *Skn*-*1* KO mice [16]. Despite unaltered food intake, *Skn*-*1* KO mice have reduced body weight with lower body fat due to increased energy expenditure. In this model, 24-h urinary excretion of catecholamines was significantly elevated, accompanied by increased fatty acid oxidation and fuel dissipation in skeletal muscle and impaired insulin secretion driven by glucose. These results suggest the existence of novel brain-mediated energy homeostatic pathways that originate from brush cells and type II taste cells in the GI tract and end in the peripheral tissues, including the adrenal glands (Fig. 1).

Parasitic infections, caused by intestinal helminths and protozoan parasites, are the most common infections in humans living in developing countries. Helminth and protozoan parasitic infections, as well as allergens, induce a type 2 immune response that involves a remodeling of the epithelial cell populations, including Goblet cell hyperplasia. Interleukin (IL)-13 is crucial for this response and is secreted by type 2 innate lymphoid cells (ILC2s) and type 2 helper T cells (TH2 cells) [17]. ILC2s are thought to be major initiators of type 2 immune responses after parasitic infections. The epithelial cytokines IL-33, thymic stromal lymphopoietin (TSLP), and IL-25 are required for the activation of ILC2s [18, 19]. Although it was assumed that the secretion of cytokines by the intestinal cells is required for type 2 immune responses following parasitic infections, it has remained elusive which types of intestinal cells initiate these responses.

Recent studies have independently reported that brush cells play a pivotal role in the initiation of type 2 immune responses [15, 20, 21]. As already mentioned, the brush cells expressing *Dclk1* and *Trpm5* constitute only a small population of the adult mouse intestinal epithelial cells [2, 3]. Using IL-25 reporter mice to identify IL-25-secreting cells, von Moltke and colleagues showed that the cells expressing *IL*-*25* are brush cells [21]. However, brush cells did not produce other epithelial cytokines activating ILC2s, including IL-33 or TSLP [21]. After helminth or protozoan infections, a hyperplasia of brush cells and Goblet cells was observed [15, 20, 21]. In addition, Howitt and colleagues reported that wild-type specific-pathogen-free mice that were bred in their facility had a larger number of brush cells than in previous reports [20]. A series of experiments using a variety of knockout or transgenic mouse strains revealed that a hyperplasia of brush cells, as well as that of Goblet cells, was dependent on IL-13 signaling produced by ILC2s. When infected with helminth, helminth expulsion was delayed in *Skn*-*1* KO mice that lack brush cells compared to wild-type mice. *Trpm5* and *alpha*-*gustducin*, which encode signal transduction molecules downstream of sweet, bitter, and umami taste receptors, are also expressed in brush cells [5]. Intriguingly, brush cell hyperplasia did not occur in *Trpm5* or *alpha*-*gustducin* KO mice [20]. In summary, brush cells are the sole source of IL-25 in the steady state. After helminth infection, IL-25 secreted by brush cells activates ILC2s, resulting in IL-13 production. IL-13 acts on intestinal stem cells and promotes the differentiation of brush cells and Goblet cells. Brush-cell hyperplasia further increases IL-25 expression and subsequent IL-13 secretion by ILC2s in a feed-forward loop.

Further studies are needed to answer the following questions: How do brush cells (which types of G protein-coupled receptors) detect intestinal parasites? Do brush cells have different roles from type 2 immune responses? What is the relationship between type 2 immune responses and energy homeostasis? The discovery of food-derived factors that regulate brush cells may open new avenues for the treatment of allergic diseases, obesity, and diabetes.

## Impact of a brown rice-derived bioactive product on feeding regulation and fuel metabolism (by Chisayo Kozuka)

### Anti-obesity and anti-diabetic components of brown rice

Previous studies have shown that a diet containing whole grains such as brown rice improves glucose metabolism compared with refined-grain diets [22, 23]. A previous clinical study of ours showed that brown rice significantly decreased body weight and ameliorated glucose and lipid dysmetabolism compared with white rice in subjects with metabolic syndrome [24]. During the refining process, most of the outer parts of the grain are removed despite their containing multiple nutrients. Dietary fiber is thought to play a pivotal role in the metabolically beneficial effects of whole grain foods. For instance, some types of fiber such as arabinoxylan and β-glucan are growth substrates for the beneficial bacteria *Bifidobacterium* and *Lactobacillus* [25, 26]. A previous study has shown that brown rice beneficially alters the composition of gut microbiota in humans [27]. These data suggest that brown rice ameliorates obesity and diabetes at least partly via its effect on the profile and activity of intestinal microbiota. Conversely, brown-rice-derived fiber has been shown to have no effect on body weight or glucose metabolism in either healthy or diabetic rats [22]. This result suggests that brown rice contains anti-obesity and anti-diabetic components other than dietary fiber. γ-oryzanol is a brown rice-specific bioactive component that is a mixture of ferulic acid esters and phytosterols such as cycloartenol, β-sitosterol, 24-methylenecycloartenol, and campesterol [7]. After oral administration, γ-oryzanol is mainly distributed in the brain, and is metabolized in the liver [28–30]. Previous studies have shown various beneficial effects of γ-oryzanol including cholesterol-lowering, anti-inflammatory, anti-cancer, anti-diabetic, and anti-oxidant activities [31]. Membrane permeability is correlated with lipophilicity, usually estimated using the logarithm of the* n*-octanol/water partition coefficient (log Po/w) [32]. The log Po/w of γ-oryzanol has been calculated as 12.1 using the XLOGP3 method [33], while that of fenofibrate, one of the most lipophilic agents among the clinically-used drugs, is 5.19 [34, 35]. This suggests that γ-oryzanol should be lipophilic enough to be membrane permeable and thus may influence intracellular metabolism. In our animal study, we demonstrated that brown rice and γ-oryzanol attenuate the preference for dietary fat and consequently decrease body weight gain [36].

### Feeding regulation and obesity

**Fig. 2:**
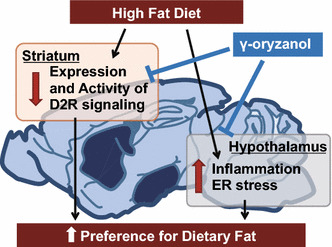
γ-oryzanol attenuates the preference for the dietary fat via both reward system and hypothalamus

Feeding behavior is regulated by the hypothalamus and mesolimbic reward system. The hypothalamic arcuate nucleus plays a key role in energy homeostasis and regulates food intake, energy expenditure, and body weight [37]. Leptin, an adipocyte-derived hormone, suppresses the appetite and increases energy expenditure through its action on the hypothalamus [38]. In obese subjects, exaggeration of inflammatory signals and endoplasmic reticulum (ER) stress induce leptin resistance in the hypothalamus and consequent hyperphagia [39–41]. Of note, we have previously shown that hypothalamic ER stress increases the preference for dietary fat [36]. When mice were allowed to choose freely between a chow and a high-fat diet (HFD), they strongly preferred the HFD. In contrast, in mice treated with 4-phenylbutyric acid, an ER stress reducer, the HFD preference was significantly decreased. These data suggest the importance of hypothalamic inflammation and ER stress as novel therapeutic targets for obesity. In an in vitro reporter assay in HEK293 cells, γ-oryzanol significantly suppressed an ER stress inducer, tunicamycin-induced activation of* cis*-acting elements such as ER stress response elements and an unfolded protein response element [36]. Therefore, we examined the possibility that brown rice and γ-oryzanol could ameliorate dysregulation of feeding. In HFD-fed mice, oral administration of brown rice or γ-oryzanol significantly decreased ER stress and inflammation in the hypothalamus [36] (Fig. 2). As expected, preference for the HFD was significantly decreased in mice treated with brown rice or γ-oryzanol [36] (Fig. 2).

Recent studies have also shown the importance of the reward system in feeding regulation [42, 43]. The reward system plays an essential role in addiction to palatable foods, which shares mechanisms with drug addiction [44]. Dopamine receptor signaling is a key component of the brain’s reward system. Dopamine release in the dorsal striatum induced by feeding is related to pleasure [45]. Reduced dopamine D2 receptor (D2R) densities in the dorsal striatum of obese humans and rodents renders them less responsive to food rewards compared with lean controls [46–48]. In humans, reduction of striatal D2R expression by the TaqIA allele of the DRD2/Ankyrin repeat and kinase domain containing 1 (ANKK1) gene locus is associated with obesity [49], while effects of weight loss after bariatric surgery are associated with elevated striatal D2R density [50]. Knockdown of striatal D2R by lentivirus-mediated short hairpin RNA rapidly induced addiction-like reward deficits and compulsion-like food seeking in rats [48]. In this context, we hypothesized that brown rice and γ-oryzanol would attenuate the preference for dietary fat at least partly through effects on D2R signaling. As expected, expression levels of striatal D2R were significantly decreased in HFD-fed mice, while γ-oryzanol restored the expression levels (Kozuka et al. manuscript under review) (Fig. 2). Anti-obesity drugs acting on the brain reward system (e.g., rimonabant) have been developed, but most were withdrawn from clinics because of considerable adverse effects including serious psychiatric problems [51]. Natural food-derived products such as γ-oryzanol may be an alternative treatment to safely regulate feeding behavior.

### D2R signaling in pancreatic islets

**Fig. 3:**
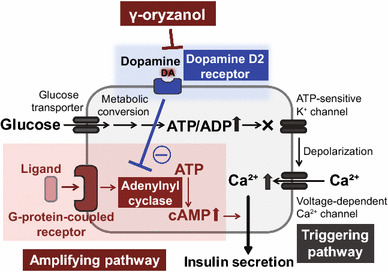
γ-oryzanol enhances GSIS via the inhibition of D2R

Recent studies have shown that molecules involved in dopamine receptor signaling are expressed not only in the brain but also in both murine and human pancreatic islets [52, 53]. In patients with Parkinson’s disease, treatment with l-DOPA, a dopamine precursor, significantly impairs glucose metabolism in a dose-dependent manner [54, 55]. In mice, D2R are confined to β-cells [52], and knockout of D2R suppresses function and replication of pancreatic β-cells during development [56]. Of note, in isolated pancreatic islets from humans, treatment with dopamine attenuates insulin via its receptors [53]. Thus, we investigated the possibility that brown rice and γ-oryzanol could ameliorate glucose dysmetabolism in HFD-fed mice via local D2R signaling in islets (Fig. 3). Insulin secretion from β-cells is mainly regulated by two distinct signaling pathways: (1) the ATP-sensitive K+ channel-dependent pathway (triggering pathway) or (2) the cAMP/PKA pathway (amplifying pathway) [57, 58] (Fig. 3). D2R signaling decreases intracellular cAMP levels in pancreatic islets as well as in the striatum and pituitary gland [59–61], and consequently decreases glucose-stimulated insulin secretion by suppressing the amplifying pathway [61]. Of note, expression levels of D2R signaling molecules were increased in islets of HFD-fed mice, and decreased with oral administration of γ-oryzanol [61]. Because γ-oryzanol decreases HFD-induced ER stress and inflammation in pancreatic islets [28], γ-oryzanol may decrease expression levels of D2R signaling molecules via the consensus elements of NF-κB in the promoter region of *Drd2* [62]. Interestingly, there were no agonist or antagonist activities of γ-oryzanol on dopamine receptors [28]. A previous study in rats suggests that γ-oryzanol may have effects on dopamine metabolism in the medial basal hypothalamus [63]. These findings suggest that γ-oryzanol has effects on local dopamine synthesis. Although further studies are necessary to fully elucidate the molecular mechanisms involved, γ-oryzanol shows potential as a treatment for obesity and diabetes.

## A novel G protein-coupled receptor-regulated neuronal signaling pathway triggers sustained orexigenic effects (by Kenichiro Nakajima)

### Introduction

Obesity has emerged as one of the most severe health problems in the world [64]. Obesity represents a major risk factor for metabolic syndrome and is associated with type 2 diabetes, cancer, and cardiovascular disease. The current obesity epidemic significantly affects public health and reduces economic activity by increasing medical costs. Owing to the low success rate of long-lasting weight loss from dietary changes and physical activity, there is a strong need for effective pharmacological strategies to stop obesity [65]. Unfortunately, the number and efficacy of appetite-suppressing drugs approved for clinical use is severely limited [65].

To develop novel appetite-suppressing drugs, it is essential to understand the neuronal circuits that regulate food intake in the brain. In this chapter, we focus on a small subpopulation of hypothalamic neurons located in the arcuate nucleus (ARC) of the hypothalamus, which synthesize and release agouti-related peptide (AgRP), a neuropeptide endowed with potent, long-lasting orexigenic activity [66]. These neurons, which are generally referred to as AgRP neurons, secrete two additional chemicals that promote acute feeding, neuropeptide Y (NPY) and GABA, a biogenic amine neurotransmitter [66, 67]. These three orexigenic agents have different sizes and effects, and, at present, their relative roles in stimulating appetite in response to different hormones or neurotransmitters are not well understood.

**Fig. 4:**
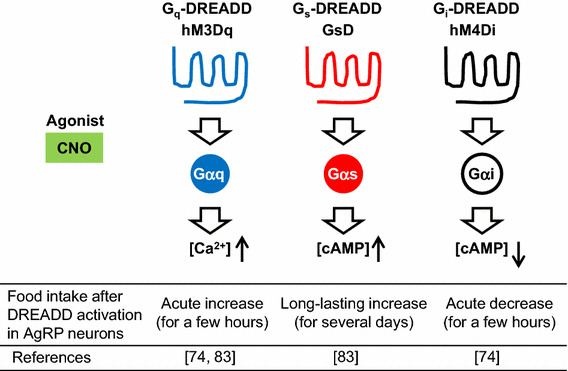
Representative DREADD receptors and their effects in hypothalamic AgRP neurons. G_q_-, G_s_-, and G_i_-type DREADDs can stimulate different signaling pathways following CNO administration. Use of DREADDs makes it possible to investigate the precise functions of specific neuronal cell types in vivo (e.g., the relationship between signaling pathways in AgRP neurons and food intake)

As indicated by the fact that AgRP neurons are next to the third ventricle and sense nutrients and hormones derived from peripheral organs, numerous studies have shown that AgRP neurons play a key role in regulating food intake and energy homeostasis [68–71]. In the past 5 years, novel toolkits in the field of neuroscience, including optogenetics and chemogenetics [72] (Fig. 4), have shown that activation of AgRP neurons leads to an acute feeding response [73, 74]. In contrast, genetic ablation of AgRP neurons in adult mice leads to a loss of appetite, and ultimately starvation [75]. This novel evidence emphasizes that AgRP neurons play a role in driving appetite.

### Use of designer G protein-coupled receptors to dissect orexigenic signaling pathways in AgRP neurons

As in the case of other cell types, the activity of AgRP neurons is regulated by cell-surface receptors belonging to the superfamily of G protein-coupled receptors (GPCRs), which are linked to distinct functional classes of heterotrimeric G proteins such as Gαq, Gαs, and Gαi [76, 77] (Fig. 4). For example, G_q_-coupled receptors can couple with G_q_, which activates phospholipase Cβ, leading to an increase in intracellular calcium ion concentration. In contrast, G_s_-coupled receptors can couple with Gαs, which activates adenylate cyclase, leading to an increase in cAMP concentrations. In contrast, G_i_-coupled receptors can couple with Gαi, which inhibits adenylate cyclase activity, leading to a decrease in cAMP concentrations. So far, the functional consequences of activating the various GPCR/G protein signaling pathways in AgRP neurons have not been systematically studied. To address this issue, we employed a new set of pharmacological tools referred to as DREADDs (designer receptors exclusively activated by designer drugs, Fig. 4). DREADDs represent mutant muscarinic acetylcholine receptors that lose the ability to bind acetylcholine or any other endogenous ligands [78]. However, DREADDs can be selectively activated by clozapine-*N*-oxide (CNO), a synthetic compound, which is otherwise pharmacologically inert [78, 79]. Currently, there are various types of newly developed DREADDs with distinct G protein-coupling properties available [80, 81]. Use of DREADDs makes it possible to monitor the in vivo consequences of activating distinct GPCR signaling pathways in a drug (CNO)-dependent fashion in specific cell types. Such studies are not possible to perform with native GPCRs, which are expressed in various tissues and cell types in general [82].

We injected Cre-dependent recombinant adeno-associated virus (AAV) containing G_s_-coupled DREADD (GsD; Fig. 4) or G_q_-coupled DREADD (hM3Dq; Fig. 4) to AgRP-ires-Cre knockin mice by stereotaxic injection. This generates mice that selectively express hM3Dq (hM3Dq-AgRP mice) or GsD (GsD-AgRP mice) in the AgRP neurons of the ARC [74, 83]. Food intake studies show that treatment of GsD-AgRP mice with a single dose of CNO resulted in an orexigenic effect that lasted for several days and was associated with a significant increase in body weight (Fig. 4, middle). In contrast, the stimulatory effect observed with CNO-treated hM3Dq-AgRP mice lasted only for a single day (Fig. 4, left), most likely reflecting differences in the cellular activity caused by the activation of G_s_- versus G_q_-dependent signaling pathways.

**Fig. 5:**
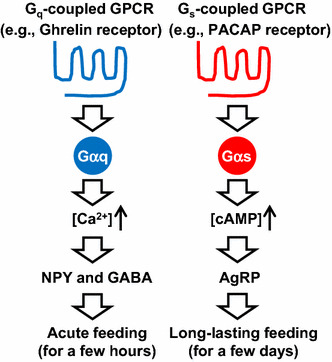
Different roles of G_q_- and G_s_-coupled GPCRs expressed by AgRP neurons in food intake. G_q_-coupled GPCRs (e.g., ghrelin receptors) stimulate the calcium signaling pathway, resulting in an acute feeding response caused by NPY and GABA release. By contrast, G_s_-coupled GPCRs (e.g., PACAP receptors) stimulate the cAMP signaling pathway, resulting in long-lasting feeding caused by AgRP release

AgRP neurons store and release the neuropeptides AgRP and NPY as well as the biogenic amine, GABA, all of which promote food intake [66]. Using a combined genetic/pharmacological approach, we demonstrated that NPY and GABA release do not contribute to the orexigenic effects mediated by GsD expressed by AgRP neurons (Fig. 5) [83]. In contrast, a GsD-mediated increase in food intake was completely blocked by intracerebroventricular treatment of GsD-AgRP mice with an anti-AgRP antibody [83]. Importantly, such effects were not seen at all in hM3Dq-AgRP mice. These data clearly indicate that the ability of G_s_-coupled DREADD to promote food intake is mediated by the release of AgRP (Fig. 5).

### Discussion

Some activators of AgRP neurons have already been revealed. As an excitatory synaptic input, glutamate activates the *N*-methyl-d-aspartate receptor, inducing neuronal excitation [84]. In terms of hormones, the stomach-derived orexigenic peptide, ghrelin, stimulates G_q_-coupled ghrelin receptors expressed by AgRP neurons to induce acute feeding as observed in hM3Dq-AgRP mice treated with CNO (Fig. 5) [74, 76, 83]. By contrast, pituitary adenylate cyclase activating peptide (PACAP) neurons in the paraventricular hypothalamus (PVH) innervate AgRP neurons that express G_s_-coupled PACAP receptors [85, 86] (Fig. 5). We found that DREADD-mediated activation of PACAP neurons in the PVH leads to long-lasting food intake, similar to that observed in GsD-AgRP mice treated with CNO [83]. This suggests that PACAP is one of the endogenous activators of AgRP neurons causing long-lasting orexigenic responses.

Recent studies have shown that when both NPY and GABA pathways are genetically impaired in AgRP neurons, activation of G_q_-coupled DREADD cannot induce an acute feeding response [67] (Fig. 5). Combined with these results, I would like to propose a working hypothesis concerning the roles of GPCRs in AgRP neurons (Fig. 5). G_q_-coupled GPCRs (e.g., ghrelin receptors) stimulate calcium signaling, resulting in an acute feeding response caused by NPY and GABA release. By contrast, G_s_-coupled GPCRs (e.g., PACAP receptors) stimulate cAMP signaling, resulting in long-lasting feeding caused by AgRP release. In the future, it will be necessary to examine when each signaling pathway is activated under normal and pathophysiological conditions in AgRP neurons.

### Conclusions

Our findings raise the possibility that drugs that block the activity of G_s_-coupled receptors endogenously expressed by AgRP neurons might become useful as appetite-suppressing drugs. The identification of the complete set of GPCRs expressed by AgRP neurons under physiological and pathophysiological conditions should provide a rational basis for this approach.

## NMDA receptor co-agonist d-serine regulates food preference (by Tsutomu Sasaki)

### Introduction

d-serine is an amino acid enantiomer of serine. Although proteins in mammals are made of the l-form of amino acids, the d-form of amino acids is present in the body. d-serine is abundant in the forebrain, and the concentration of d-serine in the brain correlates with the density of *N*-methyl-d-aspartate (NMDA) receptor [87–90]. It serves as a co-agonist for NMDA receptor and facilitates synaptic excitatory glutamatergic neurotransmission [91–93]. d-serine production and degradation are regulated in vivo by serine racemase (SR) [94–96] and d-amino acid oxidase [97], respectively. d-serine is also supplied from food, such as fermented foods, microorganisms, plants, and marine invertebrates [98]. Based on the level of d-serine present in the cerebral cortex of SR-knockout mice, it is estimated that 90 % of brain d-serine is maintained by endogenous production and 10 % is supplied from the gastrointestinal tract.

NMDA receptor signaling had been implicated in the regulation of food intake. Generally speaking, agonists for NMDA receptors were reported to suppress food intake, while inhibitors were to promote food intake [99–103]. There are two endogenous co-agonists for NMDA receptor, d-serine and glycine [91, 92, 104]. The plasma level of glycine is low in obese subjects [105], whereas it is high in anorexia nervosa patients [106, 107]. While glycine has its own cognate glycine receptors that mediate inhibitory neurotransmission [108] (instead of excitatory neurotransmission expected through NMDA receptor), and has multiple modes of biological actions outside of the nervous system, co-agonism of NMDA receptor is the only known in vivo function of d-serine [109]. Therefore, our group analyzed the effect of d-serine on feeding behavior in mice.

### Results

We first tested the effects of ad libitum drinking of d-serine water on food intake in male mice. The varying concentration of d-serine water was provided to mice fed either a normal chow diet or a high-fat diet. We found that the increasing dose of d-serine in the drinking water significantly suppressed the intake of high-fat diet, but not the normal chow diet [110]. d-serine water at 1.5 % (weight/volume) concentration also suppressed the intake of a high-sucrose diet and a high-protein diet. The effect was strong enough that some mice did not eat for 2–3 days.

In order to avoid the starvation-like response, we next gave choices of food to mice, normal chow diet and either high-fat diet, high-sucrose diet, or high-protein diet. In the presence of food choices, ingestion of d-serine reversed the preference for food in all three diets tested. d-serine altered food preferences when given simultaneously with food choices (during the acquisition phase of food preference) and when it was given after the food preference was already expressed (during the expressed phase of food preference). Upon withdrawal of d-serine water, mice re-gained the original food preference. Affecting both phases of food preferences indicated that the phenotypes are not due to food neophobia. Interestingly, the degree of effect d-serine exerts on food preference correlated with the degree of original preference for food. d-serine reversed the preference for food in the order of high-fat diet, high-protein diet, and high-sucrose diet. To prove that the effect was dependent on the co-agonism toward the NMDA receptor, we used L-701,324, the selective and full antagonist at the glycine-binding site of the NMDA receptor. L-701,324 effectively blocked the suppression of a high-fat diet preference acquisition by d-serine. These data indicated that d-serine regulates food preference through co-agonism toward the NMDA receptor.

We also analyzed the effect of d-serine on the acquisition of preference toward high-fat diet over normal chow diet in capsaicin-treated sensory-deafferented mice [111] and genetically obese *db/db* mice [112]. In both situations, d-serine prevented mice from acquiring the preference toward a high-fat diet. Interestingly, the sensory-deafferented control mice, due to the lack of sensory input, took longer time (4–5 days) to acquire the preference toward a high-fat diet, but they did acquire the preference probably relying on the post-ingestive humoral cues. Conversely, the sensory-deafferented d-serine mice acquired preference toward normal chow (instead of a high-fat diet) during the same time frame. These data indicate that the effect of d-serine on food preference is not dependent on sensory neural input (including visceral nervous input conveyed by vagus nerve) and leptin receptor signaling.

**Fig. 6:**
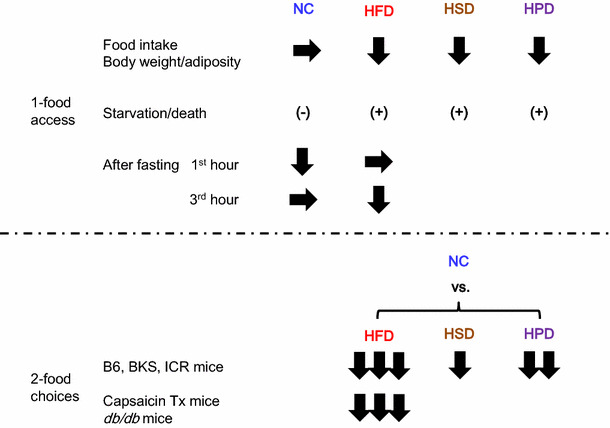
Summary of d-serine and food preference data. d-serine suppressed the intake of food that mice prefer at the given moment under 1-food access and 2-food choices

We further performed food preference experiments in the context of re-feeding after fasting because mice prefer carbohydrates over fat after fasting [113]. During re-feeding after 24-h fasting, d-serine significantly suppressed the intake of a normal chow diet but not a high-fat diet for the first hour, but suppressed the intake of a high-fat diet but not a normal chow diet by the third hour. These data indicated that d-serine suppressed the intake of food that mice find palatable at the time (Fig. 6). We also performed experiments using intraperitoneal injection of d-serine. The results are overall consistent with the finding from the oral d-serine experiments (unpublished data).

### Discussion

Animals decide whether to eat and what to eat based on the integration of peripheral sensory stimuli (such as taste and smell), internal metabolic and physiological signals that reflects the body’s needs (such as hormones and nutrients), motivation, and experience. NMDA receptor signaling contributes to both the suppression and promotion of food intake at multiple nodes of appetite, including the solitary tract nucleus, parabrachial nucleus, ventromedial nucleus of the hypothalamus, paraventricular nucleus of the hypothalamus, the lateral habenula, the lateral hypothalamic area, and the ventral tegmental area [103, 114–125]. Therefore, d-serine could affect feeding behavior at multiple aspects. So where does exogenously administered d-serine work to regulate food preference? Our experimental data collectively suggest that the main target of d-serine appear not to be at the level of sensing (both peripheral sensory stimuli and metabolic signals) or homeostatic phase, but to be at the hedonic phase controlled by the central nervous system, possibly altering the post-ingestive rewarding effect of diet. What is the implication of these results generated by the administration of pharmacological dose of d-serine to mice?

**Fig. 7:**
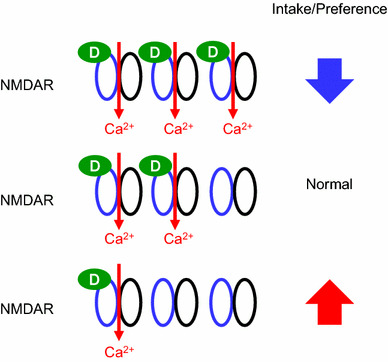
A schematic model for the relationships between NMDA-R, d-serine, and appetite. Alteration in the local d-serine concentration or sensitivity to d-serine in the neurocircuitry for feeding may affect the feeding behavior. Excessive facilitation of glutamatergic inputs through NMDA receptors by increased local d-serine concentration leads to decreases in the intake and the preference for food. Conversely, decreased local d-serine concentration may lead to increases in the intake and the preference for food

The d-serine model recapitulates some of the symptoms observed in anorexia nervosa (AN), such as the voluntary reduction in food intake despite unmet metabolic needs (observed in the 24-h fasting re-feeding experiment), avoiding foods that are considered palatable in normal condition, and the restriction of food intake accompanied by severe weight loss [126]. So hyper-activation of glutamatergic signaling through NMDA receptor may be an underlying risk factor for the pathogenesis of AN. Patients with AN exhibit abnormal reward circuitry, in both structural and functional neuroimaging studies [127, 128]. AN is highly associated with anxiety disorder comorbidities, such as obsessive–compulsive disorder and social anxiety disorder [129]. The mesolimbic mechanisms involved in hedonic food reward have been postulated to contribute to the generation of obsessive dreads in addition to obsessive desires [130, 131]. Both obsessive–compulsive disorder and AN are associated with increased glutamate levels in the cerebrospinal fluid [132, 133]. Moreover, serum d-serine levels were elevated in patients with depression [94], and the NMDA receptor antagonist ketamine showed rapid and robust antidepressant effects [134]. Glutamatergic neurotransmission through NMDA receptor, together with GABAergic neurotransmission, have been postulated to regulate the vulnerability to activity-based anorexia [135]. Our data and indirect evidence in the literature were consistent with the hypothesis that hyper-activation of NMDA receptor signaling within the central nervous system, possibly within the reward circuitry, may be one among many factors that contribute to the pathogenesis of AN. Patients with AN may have altered neurocircuitry, different sensitivities to d-serine, and/or altered d-serine metabolism (Fig. 7), which might increase their vulnerability to other factors that contribute to the pathogenesis of the disease, such as variations in brain-derived neurotrophic factor (BDNF) and adolescent stress [136]. AN has the highest mortality rate among all psychiatric disorders [137], and no randomized controlled treatment trials have demonstrated efficacy in achieving remission in adult patients with chronic AN [138]. Considering the high morbidity and the lack of effective pharmaceutical treatment, we believe that these hypotheses are worth testing.
